# Supplementary material for: BMAL1 Knockdown Leans Epithelial–Mesenchymal Balance toward Epithelial Properties and Decreases the Chemoresistance of Colon Carcinoma Cells
Source: Int J Mol Sci. 2021 May 16;22(10):5247. doi: 10.3390/ijms22105247 (PMC8157026; doi:10.3390/ijms22105247)
Supplement: Supplementary file 1 [file ijms-22-05247-s001.zip › ijms-1201936-supplementary.pdf]

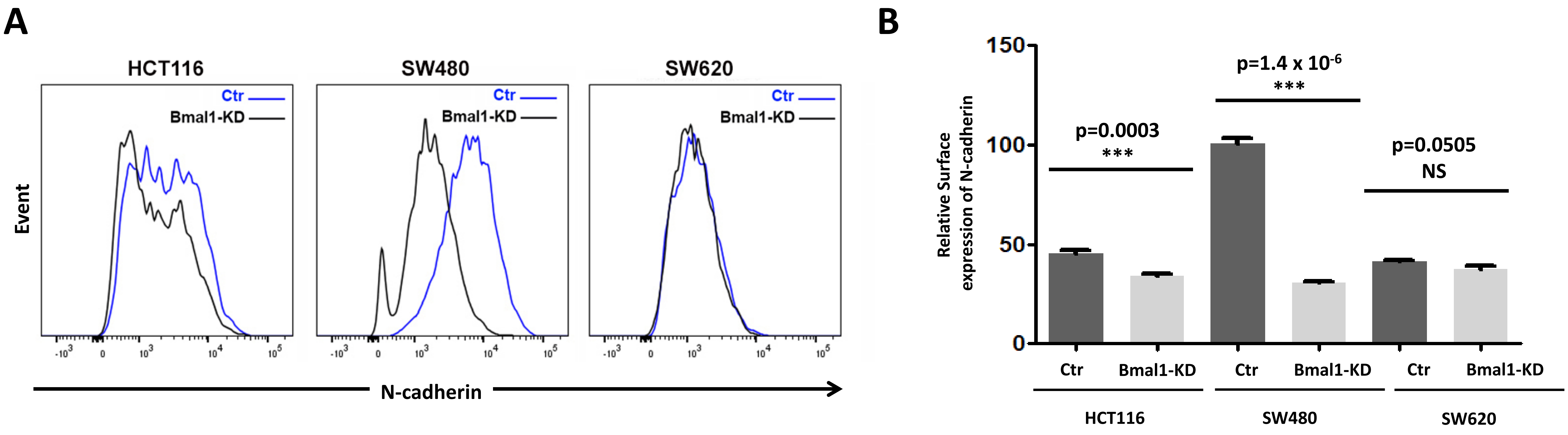

Figure S1. BMAL1-KD decreases N-cadherin plasma membrane expression of CRC cells

(**A**) A representative staining of the surface expression of N-cadherin (CD325) by flow cytometry on different BMAL1-KD (black histogram) and control (blue histogram) CRC cells. (**B**) BMAL1-KD leads to a decrease in the expression of membranous N-cadherin on the two primary CRC cell lines compared to their appropriate control cell lines (4-6 independent experiments, *p<0.05, ***p<0.001). Data are shown as means ± SEM.

Gene	Sequence	
Bmal1	FWD	AAGGATGGCTGTTCAGCACATGA
	REV	CAAAAATCCATCTGCTGCCCTG
E-cadherin	FWD	TGCCCAGAAAATGAAAAAGG
	REV	GTGTATGTGGCAATGCGTTC
N-cadherin	FWD	ACAGTGGCCACCTACAAAGG
	REV	CCGAGATGGGGTTGATAATG
Slug	FWD	GGGGAGAAGCCTTTTTCTTG
	REV	TCCTCATGTTTGTGCAGGAG
Snail	FWD	CCTCCCTGTCAGATGAGGAC
	REV	CCAGGCTGAGGTATTCCTTG
Twist1	FWD	GGAGTCCGCAGTCTTACGAG
	REV	TCTGGAGGACCTGGTAGAGG
36B4	FWD	AATCCCTGACGCACCGCCGTGATG
	REV	TGGGTTGTTTTCCAGGTGCCCTCG

Suppl. Table 1. Sequences of primers used for real-time quantitative PCR amplification
